# 2023 updated MASCC/ESMO consensus recommendations: prevention of nausea and vomiting following multiple-day chemotherapy, high-dose chemotherapy, and breakthrough nausea and vomiting

**DOI:** 10.1007/s00520-023-08224-1

**Published:** 2023-12-18

**Authors:** Bernardo Leon Rapoport, Jørn Herrstedt, Rebecca Clark Snow, Venkatraman Radhakrishnan, Mitsue Saito, Rudolph M. Navari, Teresa Smit

**Affiliations:** 1 The Medical Oncology Centre of Rosebank, 129 Oxford Road, Johannesburg, South Africa; 2 Department of Immunology, Faculty of Health Sciences, University of Pretoria, Pretoria, South Africa; 3 Department of Clinical Oncology, Zealand University Hospital, Roskilde, Denmark; 4 The University of Copenhagen, Copenhagen, Denmark; 5 Oncology Supportive Care Consultant, Overland Park, KS USA; 6 Department of Medical Oncology, Cancer Institute (WIA), Adyar, Chennai, India; 7 Department of Breast Oncology, Juntendo University School of Medicine, Tokyo, Japan; 8 World Health Organization, Birmingham, AL USA

**Keywords:** CINV, Multiple-day chemotherapy, High-dose chemotherapy, Breakthrough nausea and vomiting, NK_1_ receptor antagonists, 5-HT_3_ receptor antagonists

## Abstract

**Purpose:**

This review is an update of the MASCC/ESMO 2015 recommendations for the prophylaxis of acute and delayed nausea and vomiting induced by multiple-day chemotherapy, high-dose chemotherapy, and breakthrough nausea and vomiting.

**Methods:**

A systematic literature search was conducted using PubMed from June 1, 2015, through February 1, 2023.

**Results:**

We identified 56 references (16 were duplications or invalid), leaving 40 manuscripts for this search. The panel classified level I evidence (three manuscripts) and level II evidence (14 manuscripts). High-dose chemotherapy and stem cell transplant were discussed in four of these manuscripts, and multiple-day chemotherapy treatment in 15. Some manuscripts covered both topics. Additionally, a search for breakthrough nausea and vomiting resulted in 12 “hits.” No new relevant studies were identified.

**Conclusions:**

The recommendations for patients receiving high-dose chemotherapy with stem cell transplants and patients undergoing multiple-day cisplatin were updated. For patients receiving high-dose chemotherapy for stem cell transplant, a combination of a 5-HT_3_ receptor antagonist with dexamethasone and aprepitant is recommended. Olanzapine could be considered part of the antiemetic regimen. Patients receiving multiple-day cisplatin should receive a 5-HT_3_ receptor antagonist plus dexamethasone plus aprepitant plus olanzapine. For patients experiencing breakthrough nausea and vomiting, the available evidence suggests using a single dose of olanzapine daily for 3 days.

## Introduction

This manuscript is an update of the MASCC/ESMO 2015 recommendations for prophylaxis of nausea and vomiting induced by multiple-day chemotherapy, high-dose chemotherapy, and breakthrough nausea and vomiting [1].

## Methods

A PubMed systematic literature search was conducted for manuscripts published between June 1 2015, and February 1, 2023. Several searches were done for chemotherapy-induced nausea and vomiting (CINV) including high-dose chemotherapy and stem cell transplantation, multiple-day chemotherapy, and breakthrough nausea and vomiting. The first search consisted of (“high dose chemotherapy” or “multiple-day chemotherapy” or “stem cell transplantation”) and (emesis or CINV or “chemotherapy-induced nausea and vomiting” or nausea) and prophylaxis. The following keywords were used: (high-dose chemotherapy or multiple-day chemotherapy) and (emesis or CINV or chemotherapy-induced nausea and vomiting or nausea). A second search consisted of the following keywords: (ondansetron OR granisetron OR dolasetron OR tropisetron, OR palonosetron OR ramosetron OR azasetron or metoclopramide OR domperidone OR metopimazine OR prochlorperazine OR olanzapine OR aprepitant OR fosaprepitant OR netupitant OR fosnetupitant OR rolapitant) and (“high dose chemotherapy” or “multiple-day chemotherapy”). A third search used the keywords prophylaxis and “nausea and vomiting” and stem cell transplant. The first search resulted in 56 “hits,” the second in 16 “hits,” and the third in 41 “hits.” The search was filtered to “clinical trials.” We identified 56 references, of which 16 were duplications or invalid, leaving 40 manuscripts for this search. The panel classified these manuscripts as level I evidence (three manuscripts) and level II evidence (14 manuscripts). The 17 manuscripts were used to update the guidelines. High-dose chemotherapy and stem cell transplant were discussed in four of these manuscripts, and multiple-day chemotherapy treatment in 15. Please note some manuscripts covered both topics.

A separate search was conducted for CINV studies in germ cell tumor patients undergoing treatment with multiple-day cisplatin-based chemotherapy. In this search, only one study was identified (phase II study, a single-arm study, with a low level of evidence). This study was not used for updating the guidelines (Table 1).

**Table 1:** Prevention of nausea and vomiting following multiple-day chemotherapy, high dose chemotherapy, and breakthrough nausea and vomiting

Chemotherapy	Updated recommendations	Updated level of evidence/grade of recommendation
Multiple-day cisplatin chemotherapy		
Acute Delayed	5-HT_3_-RA + Dex + Apr + Olz^(1)^ Dex + Apr + Olz	I/A II/B for the number of days
High-dose chemotherapy for stem cell transplant	5-HT_3_-RA + Dex + Apr ^(2)^	I/A
Breakthrough	Olz^(3)^	II/B

*5-HT*_*3*_*-RA* = 5-HT_3_ receptor antagonist, *Dex* = dexamethasone, *Apr* = aprepitant, *Olz* = olanzapine

^(1^^)^The 5-HT_3_-RA should be given on the days of chemotherapy. Palonosetron could be used and should be given days 1, 3, and 5 (if 5 days of chemotherapy). Olanzapine (in a dose of 5 mg at bedtime), aprepitant, and dexamethasone should be given once daily on the days of chemotherapy and until 2 days post-chemotherapy

^(2^^)^Olanzapine could be considered part of the prophylactic antiemetic regimen. It should be used once daily at bedtime and continued for 2–3 days post-chemotherapy, as the regimen is very likely to cause significantly delayed emesis

^(3^^)^The available evidence for breakthrough nausea and vomiting suggests the use of 10-mg olanzapine, once daily for 3 days

Additionally, a search was performed for breakthrough nausea and vomiting utilizing the following keywords: (ondansetron OR granisetron OR dolasetron OR tropisetron, OR palonosetron OR ramosetron OR azasetron or metoclopramide OR domperidone OR metopimazine OR prochlorperazine OR olanzapine OR aprepitant OR fosaprepitant OR netupitant OR fosnetupitant OR rolapitant OR) and “breakthrough nausea” or “breakthrough vomiting.” The first search resulted in 12 “hits,” the search was filtered to “Clinical Trials.” None of these was relevant to the guideline update.

## Results

### High-dose chemotherapy

CINV, particularly nausea, remains a clinically significant side effect for patients receiving high-dose chemotherapy and hematopoietic cell transplant (HCT). Since the 2015 updated MASCC/ESMO consensus recommendations, most published data on CINV prophylaxis, for this population included retrospective and phase II studies. Different endpoints and small patient populations add to the challenging nature of cross-comparison of the data. The updated guidelines from the American Society of Clinical Oncology (2020) and the National Cancer Comprehensive Network (2017) included olanzapine to triple therapy as prophylaxis to CINV in high emetic risk chemotherapy, but did not address HCT in these updates. In addition, HCT patients are a heavily pre-treated and immunocompromised subgroup of patients, and some data shows the exploration of decreasing/eliminating the use of corticosteroids in these patients due to acute and chronic adverse events [2, 3].

Adding olanzapine to an NK_1_ receptor antagonist–based triplet antiemetic regimen significantly improved clinically relevant outcomes in the HCT population in a randomized, double-blind, placebo-controlled trial. Clemmons et al. [4] compared the addition of olanzapine to triplet therapy (fosaprepitant, ondansetron, dexamethasone [FOND-O]) versus triplet therapy alone (FOND) in preventing CINV in hematology patients receiving single-day and multiple-day highly emetogenic chemotherapy and HCT regimens. The cohort included 98 patients. The patients received olanzapine 10 mg (or matching placebo) on each chemotherapy day and 3 days after. Complete response (CR; no emesis and minimal nausea) was significantly higher for FOND-O in the overall (55% versus 26%, *p* < 0.003) and delayed phase (60.8% versus 30%, *p* < 0.001) but not the acute (*p* = 0.13) phase. Significantly more patients receiving FOND-O achieved no more than minimal nausea in the overall (*p* < 0.001) and delayed phases (*p* < 0.0002), as well as fewer overall mean episodes of emesis (*p* < 0.005). Within the HCT subgroup (*n* = 64), the CR, complete protection (CP; no emesis, rescue antiemetic, or significant nausea), and no significant nausea rates were significantly better for FONDO-O in the overall and delayed phases (all *p* < 0.05). The significant findings were for the autologous cohort only. No significant findings were observed in the allogeneic cohort [4].

A randomized study [3] compared the effectiveness of olanzapine plus ondansetron with palonosetron, and with ondansetron infusion for the treatment of breakthrough CINV in 62 patients undergoing hematopoietic stem cell transplantation (HSCT). The primary endpoint (no emesis, no use of rescue medication, and nausea score reduction of ≥ 50%) was achieved in 6% (ondansetron), 45% (olanzapine plus ondansetron), and 18% (palonosetron), and 6% (ondansetron), 64% (olanzapine plus ondansetron), and 18% (palonosetron) patient groups at 24 h and 48 h, respectively. This study explored the use of olanzapine for breakthrough CINV in this patient population and demonstrated it to be efficacious in this setting [3].

A retrospective study compared the effectiveness of olanzapine versus aprepitant-based regimens for CINV prevention in adult hematopoietic stem cell recipients who received high-dose melphalan. This study was not included in the guideline update because of the retrospective design. However, the results showed that olanzapine significantly reduced the number of patients who experienced acute (*p* < 0.0001) or delayed (*p* < 0.004) nausea and significantly reduced the use of rescue medications for acute-onset (*p* < 0.0046) and delayed-onset (*p* < 0.0001) CINV compared with aprepitant. Although the level of evidence of this study is low, it adds additional information to the benefit of olanzapine in this population [5].

The administration of every-other-day NEPA (fixed combination antiemetic comprised of netupitant and palonosetron) without the addition of dexamethasone (DEX) was found to be well-tolerated and very effective in controlling both emesis and nausea in patients at high risk of CINV undergoing FEAM/BEAM-based conditioning regimens. A multicenter, open-label, phase IIa study evaluated the efficacy of alternate-day dosing of NEPA administered during chemo-mobilization of 81 patients with relapsed-refractory aggressive non-Hodgkin’s lymphoma. Response rates were 77.8% for complete response (no emesis and no rescue use), 72.8% for complete control (complete response and no more than mild nausea), 86.4% for no emesis, and 82.7% for no rescue use during the overall phase (duration of chemo-mobilization through 48 h after). These phase IIa results suggest that NEPA may make it possible to eliminate corticosteroids in these heavily pretreated and immunocompromised patients [2].

A phase II study evaluated the efficacy and safety of a triple antiemetic combination of palonosetron, aprepitant, and low-dose dexamethasone in 24 multiple myeloma patients who received melphalan conditioning (100 mg/m^2^ on days 1–2) before ASCT. The authors concluded a three-antiemetic regimen consisting of palonosetron, aprepitant, and dexamethasone was safe and effective for controlling CINV due to high-dose melphalan treatment, especially during the delayed phase. Complete response (no emesis and no rescue antiemetic) and complete control (no emesis, no rescue antiemetic, and no more than mild nausea) rates were 75% and 68% during the overall phase, 88% and 86% in the acute phase, 75% and 68% in the delayed phase, and 67% and 59% in the extended phase (120–168 h), respectively [6]. This study confirms the benefit of triple therapy in the randomized phase III trial conducted by Schmitt et al. (2014). It provides evidence of clinical benefit for an NK-1RA in the extended phase up to 168 h.

The effectiveness of adding aprepitant to a standard antiemetic regimen was confirmed in patients undergoing cyclophosphamide-based conditioning regimens before hematopoietic stem cell transplant (HSCT). In a prospective, randomized, double-blind, placebo-controlled pilot study, 40 patients were randomized to receive aprepitant, compared to a placebo, in addition to ondansetron and dexamethasone for CINV prophylaxis. The average number of emesis-free days was 14.25 (SD = 1.48 days) in the aprepitant group compared to 12.45 days (SD = 2.16 days) for patients in the placebo group. Eight patients (40%) in the aprepitant group achieved CR (the absence of emesis and the absence of mild to moderate nausea) as compared to four patients (20%) in the placebo group [7].

In conclusion, controlling nausea and vomiting induced by high-dose chemotherapy and stem cell transplantation remains challenging. However, new phase III data supports considering olanzapine as an addition to triple therapy prophylaxis as part of the antiemetic regimen in managing these patients.

### Recommendation for high-dose chemotherapy and stem cell transplantation

For patients receiving high-dose chemotherapy for stem cell transplant, a combination of a 5-HT_3_ receptor antagonist, dexamethasone, and an NK_1_ receptor antagonist is recommended before chemotherapy [I, A].

#### Note

Olanzapine could be considered prophylaxis as part of the antiemetic regimen. It should be used once daily at bedtime and continued for 2–3 days post-chemotherapy, as high-dose chemotherapy is very likely to cause significant delayed emesis.

### Multiple-day chemotherapy

In the present and past, multiple-day chemotherapy studies have included drugs such as dactinomycin, dacarbazine, and ifosfamide. In the prior consensus statements, guidelines only referred to patients with germ cell tumors being treated with multiple-day cisplatin. The 2015 recommendation was a three-drug combination of a 5-HT_3_-RA plus DEX and aprepitant for the prevention of acute nausea and vomiting and DEX for delayed nausea and vomiting [1]. Previous multiple-day chemotherapy studies were incorporated in this review, as well as newly published data, including a large phase III study [8].

The OFFER study [8] was a randomized, double-blind, placebo-controlled phase 3 trial conducted in 22 hospitals in China. Eligible patients were between 18 and 75 years old and had the diagnosis of malignant solid tumors. The patients in this study received various chemotherapy containing 3-day cisplatin (3-day total dose ≥ 75 mg/m^2^). Enrolled patients were randomized to receive either 5-mg olanzapine or placebo orally before bedtime for 5 days in combination with intravenous fosaprepitant at a dose of 150 mg given 1 h before the administration of cisplatin on day 1, ondansetron intravenously at a dose of 8 mg, and dexamethasone orally at a dose of 6 mg administered 30 min before cisplatin from days 1 to 5. In total, 349 patients were included in this study. There were 175 patients randomly allocated to receive olanzapine and 174 who received placebo. A CR was defined as no vomiting or rescue medication from days 1 to 8 following chemotherapy administration. The proportion of patients who attained a CR in the overall phase was significantly higher in the olanzapine group than in the placebo group (69% compared to 58%, *p* = 0.031). A CR was documented in the olanzapine group compared to the placebo group in most subgroups. Four factors were significantly associated with CR in a multivariate analysis: the treatment groups, gender, the baseline plasma concentration of 5-HT, and prior radiotherapy. Reported adverse events associated with olanzapine were all grade 1 or grade 2. Insomnia was less frequent with olanzapine than with placebo (1% versus 6%, *p* = 0.020), whereas dry mouth was more frequent with olanzapine (6% versus 1%, *p* = 0.035). The authors concluded that olanzapine administered at a dose of 5 mg in combination with fosaprepitant, ondansetron, and dexamethasone was superior to triple antiemetic therapy alone for patients receiving multiday cisplatin-based chemotherapy regimens. Based on these results, the four-drug combination should be recommended as the best antiemetic regimen for patients receiving multiday cisplatin-based chemotherapy. Additionally, the authors suggested that a baseline plasma concentration of 5-HT (an exploratory endpoint) may be used to identify individuals predisposed to CINV; however, this suggestion requires additional confirmatory research.

A second study investigated olanzapine in a dose of 5 mg daily for 4 days compared to aprepitant 125 mg day 1 and 80 mg daily days 2–3 both combined with the 5-HT_3_ receptor antagonist, tropisetron, 5 mg i.v. daily days 1–3, and i.v. dexamethasone daily days 1–3 (10 mg in the olanzapine group and 5 mg in the aprepitant group [9]. This was a prospective randomized controlled trial in patients treated with cisplatin-based chemotherapy at a dose of 25 mg/m^2^ per day for 3 days. The primary endpoints were total protection (TP; no vomiting symptoms during the overall phase and on the 100 mm nausea research score table, the maximum value of the nausea score is 25 mm) during the acute phase (AP) (0–24 h), delayed phase (DP) (25–120 h), and overall phase (OP) (0–120 h) between the two groups. The secondary endpoints were the complete response (CR) and total control (TC) during the three phases. The Kaplan–Meier curve and log-rank test were also used to compare the time to the first vomiting episode. TP rates were similar for the olanzapine and aprepitant groups. For the AP, they were 94% (98/104) compared to 95.45% (98/106) (*p* = 0.61). For the DP, they were 54% (57/104) compared to 54% (58/106) (*p* = 0.99). For the OP, the values were 53% (58/105) compared to 55% (56/104), respectively (*p* = 0.99). No significant differences were detected between the time of the first vomiting event when comparing the two groups. The authors concluded that olanzapine at a dose of 5 mg was equivalent to aprepitant. Unfortunately, methodological errors included (i) the fact that the study was underpowered, (ii) the authors did not state the calculation of the sample size, (iii) the study was designed for non-inferiority, and (iv) the fact that TP rates are not the standard way of assessing the primary efficacy endpoint in antiemetic studies [9].

NEPA, combined with dexamethasone, was investigated in soft tissue sarcoma patients treated with multiple days of epirubicin and ifosfamide chemotherapy. The primary endpoint of this single-arm study (phase 2) was CR, defined as no emesis or rescue medication during the overall phase (0 to 120 h in cycle 1). Secondary endpoints were CR during the overall phase of cycles 2 and 3. The primary endpoint was reached in 88.9% of patients. The CR rates in cycles 2 and 3 were 88.9% and 82.4%, respectively. The antiemetic regimen was well tolerated. The authors concluded that this phase 2 study demonstrated the benefit of one shot of NEPA to prevent CINV in sarcoma patients receiving multiple-day chemotherapy treatment. This study was not included in the guideline update due to the sample size (pilot study) and the single-arm design [10].

Multiple-day chemotherapy is also used in the treatment of patients with hematological malignancies. The Rete Ematologica Pugliese group conducted a phase II multicenter study comparing palonosetron with aprepitant to palonosetron alone in patients undergoing different induction chemotherapy regimens to treat acute myeloid leukemia (AML) [11]. Patients were randomly assigned to palonosetron (0.25 mg) every other day until the last dose of chemotherapy alone or palonosetron in combination with aprepitant given on days 1–3. Chemotherapy for leukemia consisted of an anthracycline on days 1 to 3 plus cytarabine administered for 5 to 10 days. The primary endpoint was CR (no emesis or rescue medication) over the whole study period (days of chemotherapy plus two additional days). The study enrolled a total of 134 patients (130 were evaluable). Sixty-eight patients were treated with palonosetron and aprepitant, and 62 received palonosetron alone. This study omitted the use of corticosteroids. The primary endpoint of CR was not attained. The CR rates were similar between the two treatment arms (72% compared to 69%; *p* = 0.55). However, a higher proportion of patients treated with palonosetron in combination with aprepitant were free from nausea during the entire study period (43% compared to 27%; *p* = 0.03). There was also a significant difference in favor of the two-drug regimens in the unplanned endpoint of antiemetic treatment failure (median, 5 days vs 3 days; *p* = 0.03). This endpoint consisted of first CINV event. The authors concluded that this study suggests that every‐other‐day palonosetron plus 3‐day aprepitant can benefit clinical control of CINV caused by multiple days without using corticosteroids in treating AML. However, this study has several methodological shortcomings, including lacking a corticosteroid control arm, sample size, assessing nausea, and using different AML regimes [11].

A breast cancer study used an open, randomized design and investigated the safety and efficacy of the NK_1_ receptor antagonist, aprepitant (once daily, days 1–3), as part of a combined antiemetic prophylactic regimen (oral tropisetron once daily, days 1–2, and oral dexamethasone once daily, days 1–4) in the prevention in patients treated with multiple-day anthracycline-based chemotherapy (*n* = 100). The complete response for the overall, acute, and delayed phases were statistically different between the aprepitant group and standard group (80% compared to 48%, *p* = 0.001; 92% compared to 74%, *p* = 0.017; and 80% compared to 48%, *p* = 0.001), respectively. The aprepitant group had a longer time to the first event of emesis than the standard group (tropisetron and dexamethasone). The authors concluded that adding aprepitant therapy is efficacious and safe in the multiple-day anthracycline chemotherapy-induced nausea and vomiting control. Methodological errors in this underpowered study included the sample size and the fact that most cancer centers worldwide do not routinely use multiple days of anthracycline chemotherapy to manage breast cancer [12].

Of paramount importance is the use of regimens with lower doses of corticosteroids as a significant concern is potential late toxicity development. It has been previously established that 9% of patients receiving multiple-day cisplatin for metastatic testicular cancer developed avascular necrosis of the hip [13].

### Recommendation for multiple-day cisplatin chemotherapy

Patients receiving multiple-day cisplatin should receive a 5-HT_3_ receptor antagonist (once daily on the days of chemotherapy) plus dexamethasone (once daily from day 1 and until 2 days post-chemotherapy) plus aprepitant (125 mg orally on day 1 and 80 mg orally from day 2 and once daily until 2 days post-chemotherapy) plus olanzapine (5 mg once daily from day 1 and until 2 days post-chemotherapy) for acute and delayed nausea and vomiting (I, A, but II, B for the number of days).

#### Note

Palonosetron could be used and should be given days 1, 3, and 5 (if 5 days of chemotherapy). Olanzapine should be given at bedtime.

### Breakthrough nausea and vomiting

Breakthrough nausea and vomiting remains a significant clinical problem. Breakthrough nausea and vomiting is defined as nausea and vomiting during chemotherapy regardless of prophylaxis with guideline-directed antiemetics. An observational, prospective, multi-center study analyzing data from 1910 Japanese patients scheduled for moderately or highly emetogenic chemotherapy (MEC and HEC, respectively) showed almost half of the patients experienced breakthrough nausea and vomiting despite prophylactic use of antiemetics [14]. Prior history of CINV significantly predicts CINV in subsequent treatment cycles [15]. To prevent nausea and emesis, agents utilized in managing CINV, should be given as primary prophylaxis before starting chemotherapy. In most studies, CR is defined as no emetic episodes and no use of rescue medication. Some studies separately incorporate a VAS with ratings of zero (no nausea) to 100 mm worse possible nausea) in an attempt to assess nausea as a separate entity and exploratory endpoint. Currently, nausea, during chemotherapy and for several subsequent days, rather than emesis, is the most prevalent problem. Furthermore, VAS merely provides a qualitative assessment of nausea and fails to consider the duration of nausea. Some studies also evaluate the patient’s quality of life in addition to quantifying nausea and vomiting.

Clinicians should be aware of additional causes of persistent nausea and vomiting besides chemotherapy. Differential diagnoses include, but are not limited to, gastrointestinal (antiemetic-related constipation, digestive mucositis, hepatic metastases), neurological (CNS metastases), metabolic (dyselectrolytemia), drug (narcotics, antibiotics), and psychophysiological causes. In addition to managing CINV, clinicians should address and treat the above clinical complications.

### Treatment of Breakthrough CINV

As mentioned above (see high-dose chemotherapy), a randomized study by Nakagaki et al. compared the effectiveness of olanzapine (10 mg once daily) plus ondansetron with palonosetron, and with ondansetron alone for the treatment of breakthrough CINV in 62 patients undergoing hematopoietic stem cell transplantation (HSCT). Olanzapine plus ondansetron was superior to ondansetron alone and to palonosetron [3].

A single-arm, prospective trial analyzed the total control (no emesis, no nausea, no rescue medications) (primary endpoint) and early efficacy using the nausea scores at 30, 60, and 120 min after taking olanzapine (secondary endpoint) in 19 patients who took olanzapine (5 mg daily for 3 days) for breakthrough CINV after carboplatin-based therapy [16]. Even though nausea was significantly reduced after 30 min (*p* = 0.0078), and the scale had been reduced by 67% from the baseline after 60 min, the olanzapine 5 mg did not show the expected effect on the complete disappearance of CINV within 24 h. The main methodological problems with this study are the endpoints that were not validated, as well as the phase II study design.

A well-conducted meta-analysis was performed to identify randomized controlled trials comparing olanzapine to other standard antiemetics for either prevention or rescue [17]. In the breakthrough setting, olanzapine was statistically and clinically superior in the “no emesis” endpoint analyzed. Given the possible reduction in side effects, a 5-mg dose of olanzapine could be considered. Additional research is required to establish the optimum dose of olanzapine in this clinical setting.

In a single center, phase II study, 80 patients were analyzed to investigate the effectiveness of oral aprepitant as a second rescue if the primary rescue failed. Seventy-six percent of the patients failed the first rescue. This study was considered negative, as only 16.3% of the patients satisfied the successful rescue criteria (no vomiting and no need for additional rescue therapy, with nausea up to grade 1) [18].

### Recommendation for Breakthrough CINV

The available evidence for breakthrough nausea and vomiting suggests the use of olanzapine if not used for primary prophylaxis (some evidence supports a single daily dose of 10 mg for 3 days) (II, B).

## Conclusions

Only a few studies have been published investigating the prophylaxis of acute and delayed nausea and vomiting induced by high-dose chemotherapy, multiple-day chemotherapy, and breakthrough nausea and vomiting since the 2015 consensus conference. An important advance in the last 5 years regarding managing these patients is the addition of olanzapine to improve CINV prevention for patients receiving high-dose chemotherapy and autologous stem cell transplants. This agent was also efficacious in patients receiving multiple-day cisplatin-based chemotherapy regimens. Olanzapine has been incorporated into the treatment of breakthrough nausea and vomiting. However, in this clinical setting, there are currently no new recommendations. While prevention of vomiting is relatively well managed, nausea remains an unmet medical need. Future studies should focus on improving nausea control in these three patient subsets. The optimal dose and schedule of olanzapine remain unestablished, and randomized trials should be conducted for this agent to determine maximum efficacy and minimal side effects.
